# Perinatal Imaging in Partnership with Families (PIPKIN): Longitudinal cohort study protocol

**DOI:** 10.1371/journal.pone.0330915

**Published:** 2025-12-30

**Authors:** Kaili Clackson, Dianna Ilyka, Borja Blanco, Staci Meredith Weiss, Isobel Greenhalgh, Maria Rozhko, Laura Carnevali, Matthew Weatherhead, Xiangyi Ma, Beyza Ustun-Elayan, Ezra Aydin, Noelia Corral Ferre, Topun Austin, Nadja Reissland, Mark H. Johnson, Sarah Lloyd-Fox, and the PIPKIN Study Team

**Affiliations:** 1 Department of Psychology, University of Cambridge, Cambridge, United Kingdom; 2 Department of Forensic and Neurodevelopmental Science, King’s College London, London, United Kingdom; 3 Centre for Research and Psychological Wellbeing, University of Roehampton, London, United Kingdom; 4 Rosie Hospital, Cambridge University Hospitals Foundation Trust, Cambridge, United Kingdom; 5 Department of Psychology, Durham University, Durham, United Kingdom; PLOS: Public Library of Science, UNITED KINGDOM OF GREAT BRITAIN AND NORTHERN IRELAND

## Abstract

While advances in behavioural and neuroimaging methods suitable for use with infants have greatly increased our understanding of infant brain function, cognition and behaviour in recent years, relatively little is known about the rapid period of development during the last trimester of pregnancy and first weeks and months after birth, as well as the roles that the social environment and stress play in shaping this development. This protocol paper outlines The UK Perinatal Imaging in Partnership with Families (PIPKIN) Study, a unique, multi-method, longitudinal cohort study investigating the early development of fetal and infant neurocognitive function and behaviour, and how the infant’s social and family environment shapes this development. The study follows families from a range of socio-economic backgrounds who participate at ten timepoints, from the third trimester of pregnancy until their infant is nine months old, with three visits taking place during the infant’s first postnatal month. The study harnesses recent methodological advances coupled with the drive for more ecologically valid data collection by undertaking many of these visits in families’ homes. Methods include measures of fetal behaviour using 4D ultrasound scanning; infant brain imaging using fNIRS and EEG; a full-day video recording of the home environment from the infant’s perspective, with physiological measures; measures of recent stress in both infant and mother; questionnaires relating to the home environment as well as parents’ feelings, attitudes, health and parenting routines; and standardised measures of infant behaviour and development. Specific aims are to investigate: i) individual differences in basic sensory, behavioural and motor processing between late prenatal and early postnatal periods; ii) rapid change in cortical functions over the first month, particularly for brain networks that support social behaviour; iii) effects of social interaction on developing brain function; and iv) individual differences in developmental trajectories associated with poverty-related contextual factors.

## Introduction

### Background & rationale

Birth represents the most significant environmental change that a brain experiences in its lifetime [1,2], and triggers a period of rapid growth and development, making the late prenatal and the early postnatal period a critical time for identifying early-stage markers of later outcomes. While the immediate postnatal period has been little studied, recent evidence supports its importance as: (a) interneurons are still migrating to the prefrontal cortex [3]; (b) initial studies indicate rapid change in brain function over the first days post-birth [4]; and (c) brain development atypicalities can first emerge during the first six months of life [5,6].

Preparation for this new world outside of the womb begins during pregnancy [7], and recent advances in 4D ultrasound technology have allowed researchers to measure fetal behaviour and development from the second trimester onwards [8,9], allowing for the assessment of transnatal sensory continuity from pre-to-postnatal life [10,11]. From the end of the second trimester, when fetal sensory systems are developed and fetuses can sense, distinguish, and react to various stimuli such as touch, taste, smell, light, and sound [12], measurements of fetal behaviours in response to sensory stimuli and assessment of fetal learning and memory through habituation [13–16] are possible.

Relatively little is known about the rapid period of development in the first weeks and months after birth, partly due to this window of development coinciding with a challenging time for families to travel and participate in research outside of their community and home. Therefore, it is critical that we find better ways to bring research to homes and communities rather than expecting families to come to us. Furthermore, sampling in the home allows us to unravel developmental effects associated with the child’s environment. Advances in portable/wireless functional Near Infrared Spectroscopy (fNIRS) and electroencephalography (EEG) technology mean that there are now suitable and reliable ways to study brain structure and function in newborns, allowing for the investigation of rapid postnatal brain specialisation through repeated testing of infant cortical responses from birth, while the miniaturisation of audio-visual and physiological recording devices allows naturalistic recordings of an infant’s everyday experiences at home.

While such new methodologies have led to advances in our understanding of brain and cognitive development over the last decade, this research has usually been carried out in Western societies with financially mobile participants within relatively high-income settings [17], while those most at risk for experiencing socio-economic and health challenges are far less likely to participate in research [18]. Despite this, recent large-scale studies in high-income countries have shown links between growing up in poverty and childhood brain development [19–24]. For example, Noble et al [23] showed that higher parental education levels, and greater family income predict greater brain surface area in the child, with the effects seen most strongly in the most disadvantaged children [for a review of studies of brain volume and poverty in infancy see 25]. Such findings have led to models linking specific components of socioeconomic status (SES) to structural and functional brain development and life outcomes [26,27]. However, many of these studies are based on correlational analyses in later childhood rather than on observing longitudinal brain development from prenatal life to infancy to fully understand the mechanistic processes driving these differences as they unfold. Investigations of brain function and anatomical specialisation potentially offer an opportunity to identify atypicalities and effects of deprivation much earlier, allowing for earlier and more effective interventions [28].

Clearly, the social and physical environment in which a child grows up has profound effects on both brain development, and their ability to lead a healthy and productive life [29,30]. A growing body of research highlights the importance of social relationships in the development of the infant brain [31,32] yet few studies have investigated how measures of brain function relate to an infant’s everyday experiences of social interactions at home. Stress is also known to have a profound influence on development [33,34], with even relatively mild stressors such as environmental noise affecting the development of attention and emotion regulation measured via cardiac responses and language development [35,36].

Maternal psychosocial stress during pregnancy is a multifaceted phenomenon that impacts maternal emotions, behaviour, and physiology, potentially altering fetal physiology and behaviour through various pathways [37–39]. This complex interaction sets the trajectory for fetal programming of the HPA axis as evidenced by high levels of cortisol crossing the placenta in the early stages of fetal development, before infants have functioning endocrine systems in place to produce their own glucocorticoid [40,41]. Prior literature [42] suggests that a mother’s pervasive stress and chronic immune responses result in the suppression of infant glucocorticoid production in-utero. The higher threshold of stress required to activate an infant’s HPA axis can persist after birth, such that it appears infants maintain their adaptation in-utero by balancing their own production with their mother’s high level of production [43]. Furthermore, a study found that the maternal cortisol awakening response during pregnancy significantly predicts infants’ cortisol and behavioural responses to stressors at 9 months of age [44]. As circulating cortisol binds with keratin elements found in bio samples, fingernails can be assayed to provide a trace of stress over several weeks and hair can be assayed to provide a trace of longer-term stress exposure over several months [45,46].

### The PIPKIN study

The PIPKIN Study (Perinatal Imaging in Partnership with Families) is a unique, comprehensive, longitudinal study investigating typical development of the infant brain and the impact of the infant’s social and family environment, following families from a diverse range of socio-economic backgrounds starting in the third trimester of pregnancy, until the infant is 9 months old. Antenatal recruitment of the full cohort of 130 participant families is nearly complete, and data collection is ongoing.

**Fig 1:**
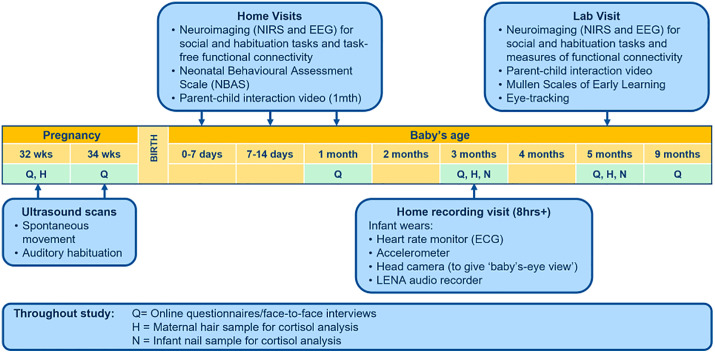
The PIPKIN study data collection timeline from pregnancy through to nine months of age.

We use 4D ultrasound scans to measure the fetal behaviour during the third trimester; brain imaging techniques (NIRS, EEG) to assess rapid specialisation of brain function over the first weeks of life; measures of current stress in both infant and parents; questionnaires relating to the home environment as well as parents’ feelings, attitudes and parenting routines; and standardised measures of behaviour and development across the first weeks and months of life. The study involves two antenatal ultrasound visits at 32 and 34 weeks’ gestation, followed by intensive measures during the immediate postnatal period, with two home research visits scheduled during the first two to three weeks and another at one month after birth. Infants visit the Babylab for a further research visit at 5 months. As well as experimental testing sessions, when infants are three months of age, we also record the infant’s home environment for a full day to gain an understanding of each infant’s everyday social interactions. Families are followed until their infant reaches 9 months of age. The complete study timeline can be seen in Fig 1.

Throughout the study, parental and infant stress is indexed through hair and fingernail bio samples, as well as questionnaires (i.e., the Perceived Stress Scale).

Although other studies have collected neural data during the first six months of life, to our knowledge no longitudinal studies have included fetal behavioural measures continuing into the early postnatal period as extensively, with multiple technologies for assessing brain function, behaviour, cognition and environmental contextual factors. This detailed data set will form an essential reference for future work on infant development, informing future studies identifying infants whose development may need additional support due to factors such as socioeconomic circumstances, and forming the basis for effective and targeted intervention strategies.

This protocol paper explains the methods and tasks used in the study, as well as future plans for data analysis.

### Aims and hypotheses

The primary overall aim of the study is to investigate the development of neurocognitive function, particularly related to the processing of social information and family context. This study investigates four novel hypotheses about developmental changes and emerging brain functions from the third trimester to around 9 months postnatal (Table 1).

**Table 1:** Study hypotheses.

Hypotheses:		Assessed by:
1)	Inter-individual differences in basic sensory and motor processing are consistent between late prenatal and early postnatal periods.	Consistency between corresponding measures observed in the third trimester and those in the early postnatal period.
2)	There is rapid change in cortical functions over the first postnatal days, particularly in brain networks that support social behaviour.	Changes between brain function measurements at around one week, two weeks and one month.
3)	Development in brain function is associated with experience of other humans in social interaction.	Brain function measures and social experience indexes in the home environment from birth to three months.
4)	Individual differences in developmental trajectories are associated with low resource (poverty) associated contextual factors	Assessed by relating measures of brain function, stress, and behavioural outcomes to questionnaire data measuring factors associated with low resource settings.

Additionally, the wide range of measures collected during the study will also allow us to explore further hypotheses related to specific measures including parental mental health measures, language use in the home, caregiving, maternal cortisol levels during pregnancy, maternal diet, infant behaviours, regulation and organisation, and brain development.

## Methods and materials

### Recruitment and eligibility criteria

Families are recruited from Cambridge (UK) and surrounding villages, either via the Rosie Maternity Hospital where patients attending for routine 20-week ultrasound scans are given information about the study, or from the community via flyers and social media advertisements. To ensure the participant sample includes families from a range of socio-economic backgrounds, community and hospital recruitment is targeted at participants living in more deprived areas, as identified by the Indices of Multiple Deprivation. Inclusion criteria for the study are shown in Table 2, with criteria relating to the infant re-assessed at birth.

**Table 2:** Inclusion criteria.

Mother	Infant
• 18 years old or older • Singleton healthy pregnancy • English language level adequate to answer questionnaires and communicate with research team.	• Born between 37–42 weeks gestation • Birthweight >2.5 kg • No neurological deficits or developmental disorder diagnosed during pre/postnatal checks.

Father/partner involvement is optional but encouraged, involving completing interviews/ questionnaires and taking part in recorded parent-child interaction (PCI) sessions when available. As currently in the UK partners are less likely to be available during the first months of parental leave, we prioritise the collection of maternal data for these variables and collect father/partner data second. Participant families receive a copy of the two 4D ultrasound scans as well as gift vouchers and small gifts during the study as an incentive to continue.

Piloting of aspects of the protocol took place from February 2022 with 15 families. Recruitment to the study commenced on 1^st^ June 2022.

125 families have been recruited into the study so far. Of these, forty-one have withdrawn during the study, and there may be further withdrawals among those currently enrolled. Withdrawals primarily occur around the time of the infant’s birth (i.e., after ultrasound visits). Reasons given for withdrawals included family needs, the challenges of coping with more than one child, and time constraints.

Due to the high withdrawal rate, the original recruitment target of 120 infants has been extended to allow for further postnatal recruitment until 1^st^ November 2025, with the intention of achieving a minimum of 80 datasets relating to each study hypothesis. Data collection for all time points will conclude by 1^st^ March 2026.

Parents provide written consent for the full study on behalf of themselves and their (unborn) infants before any study data is collected. Written consent is provided either on a paper consent form or via an electronic online consent form, and consent is obtained either at the start of the first ultrasound visit or prior to this. Where written consent is provided at the first ultrasound visit, this is witnessed by a member of the research team. Consent always takes place after the participant has been provided with full details of the study and the opportunity to ask questions. Verbal consent is then confirmed by researchers at all study visits. If consent is not given, the visit is terminated and this is documented in study paperwork.

### Study design

PIPKIN participants attend study visits at seven timepoints as shown in Fig 1. Families were consulted on all aspects of the research during the piloting phase and protocols were adjusted in response to parent feedback.

Written ethical approval for the study was obtained from Cambridge Psychology Research Ethics Committee (Application no. PRE.2020.123) and the East Midlands – Nottingham 1 NHS Research Ethics Committee (REC reference 22/EM/0004, IRAS project ID: 295027).

Further details of measures taken across timepoints are given in Table 3 and elaborated below. Further details of each paradigm will be added to the Open Science Framework PIPKIN protocol paper page.

**Table 3:** Summary of the PIPKIN protocol.

	Antenatal		Postnatal							
Ultrasound measures	32 wks	34 wks	0-7 days	7-14 days	1 mo	2 mo	3 mo	4 mo	5 mo	9 mo
**Fetal biometric measures**	**x**	**x**								
**Spontaneous (baseline) movements**	**x**	**x**								
**Social/Non-social auditory habituation task**	**x**	**x**								
**Neuroimaging measures**	**32 wks**	**34 wks**	**0-7 days**	**7-14 days**	**1 mo**	**2 mo**	**3 mo**	**4 mo**	**5 mo**	**9 mo**
**fNIRS: Social/Non-social visual only (awake)^**			**x**	**x**	**x**					
**fNIRS: Social/Non-social auditory only (asleep)**			**x**	**x**	**x**					
**fNIRS: Social/Non-social visual and auditory (awake)**									**x**	
**fNIRS: Habituation and novelty detection (HAND) (asleep, awake at 5mths)**			**x**	**x**	**x**				**x**	
**fNIRS: Task-free functional connectivity (asleep)**			**x**	**x**	**x**					
**fNIRS: Functional connectivity (awake)**									**x**	
**EEG: Auditory Oddball Habituation (asleep)**					**x**				**x**	
**EEG: Resting state (asleep)**					**x**				**x**	
**EEG: Gaze ERP task (awake)**									**x**	
**Behavioural/Neurocognitive measures**	**32 wks**	**34 wks**	**0-7 days**	**7-14 days**	**1 mo**	**2 mo**	**3 mo**	**4 mo**	**5 mo**	**9 mo**
**Neonatal Behavioural Assessment Scale (NBAS)**			**x**	**x**	**x**					
**Parent-Child Interaction video (PCI)**					**x**				**x**	
**Mullen Scales of Early Learning (MSEL)**									**x**	
**Eye-tracking: Gap/Overlap (disengagement)**									**x**	
**Eye-tracking: Non-social contingency**									**x**	
**Eye-tracking: Fifty faces**									**x**	
**Eye-tracking: Dancing ladies**									**x**	
**Home Environment Measures (8hr+ recording)**	**32 wks**	**34 wks**	**0-7 days**	**7-14 days**	**1 mo**	**2 mo**	**3 mo**	**4 mo**	**5 mo**	**9 mo**
**Electrocardiogram (ECG)**							**x**			
**Accelerometry**							**x**			
**Baby head-camera recording (baby’s eye view) and room camera**							**x**			
**LENA language environment assessment**							**x**			
**Questionnaires/Interviews – Family**	**32 wks**	**34 wks**	**0-7 days**	**7-14 days**	**1 mo**	**2 mo**	**3 mo**	**4 mo**	**5 mo**	**9 mo**
Demographic and Family Unit Information*	**M**								**M**	
**General Household Information**	**M**									**M**
Parental Mind-Mindedness interview*	**M/P**								**M/P**	
**Expectations about your child**	**M/P**									
**MacArthur Subjective Social Status Scale (SSSS)**	**M**									**M**
**Bringing order out of chaos**	**M**				**M**				**M**	**M**
**Adverse Childhood Experience (ACE) Questionnaire**							**M**			
**Brief form of the perceived social support questionnaire**	**M**				**M**					**M**
**Social Exposure**	**M**				**M**	***M***	**M**	**M**		**M**
**Positive and Negative Affect Schedule (PANAS)**	**M/P**	***M/P***			**M/P**				**M/P**	**M/P**
**Perceived Stress Scale (PSS)**	**M/P**	***M/P***			**M/P**				**M/P**	**M/P**
**Hospital Anxiety and Depression Scale (HADS)**	**M/P**				**M/P**				**M/P**	**M/P**
**Pregnancy Related Anxiety Scale**	**M/P**	***M/P***								
**Parental Stress Scale**								**M/P**		**M/P**
**Comprehensive Parenting Behaviour Questionnaire**								**M/P**		**M/P**
**Maternal Confidence Questionnaire**								**M/P**		**M/P**
**Adult Temperament Questionnaire (short form)**		***M/P***								**M/P**
**Expectations About Your Child Questionnaire**	**M/P**									
Ordinariness of the day Questionnaire*			**M**	**M**	**M**				**M**	
**Questionnaires/Interviews – Infant/Child**	**32 wks**	**34 wks**	**0-7 days**	**7-14 days**	**1 mo**	**2 mo**	**3 mo**	**4 mo**	**5 mo**	**9 mo**
**Feeding Questionnaire**					**M**	***M***	**M**	**M**		**M**
**Sleep Questionnaire**					**M**	***M***	**M**	**M**		**M**
**Sleep Questionnaire – shortened partner version**					**P**	***P***	**P**	**P**		**P**
**Infant’s life and routines**					**M**				**M**	**M**
**Infant Behavioural Questionnaire (IBQ) very short form**									**M**	**M**
**Infant/toddler Sensory Profile (ITSP)**									**M**	**M**
**Oxford Communication Development Inventory (CDI) (UK English)**										**M**
**Vineland (caregiving)**										**M**
**Medical details/sample collection**	**32 wks**	**34 wks**	**0-7 days**	**7-14 days**	**1 mo**	**2 mo**	**3 mo**	**4 mo**	**5 mo**	**9 mo**
Healthy Pregnancy Questionnaire*			**M**							
**Antenatal Hospital Visit Questionnaire**	**M/P**									
**Maternal Diet Diary (Intake24UK)**		**M**			**M**					**M**
**Hair sample – cortisol analysis (M)**	**M**								**M**	
**Fingernail sample – cortisol analysis (I)**							**I**		**I**	

*Information collected verbally before (via phone calls) or during visit. All other questionnaires answered online (via REDCap) at home.

^ This paradigm was not run for the full length of the study, see fNIRS section below.

Entries in italics indicate where questionnaires were initially completed, before the questionnaire timetable was simplified to reduce the time burden on families in February 2024. From February 2024, all the online questionnaires from 32 weeks to 5 months were collected in-person on an iPad during visits.

fNIRS = functional near infrared spectroscopy; EEG = electroencephalography. For questionnaires/interviews/cortisol samples, letters show who data is collected from: M = mother, P = father/partner, I = infant.

### Ultrasound measures

In the last trimester of pregnancy, antenatal scans are recorded at 32- and 34-weeks gestation using a GE Voluson E10 ultrasound machine, equipped with the GE eM6C matrix electronic 4D probe, at the Rosie Maternity Hospital in Cambridge. During this session, the sonographer first completes fetal anthropometric measures such as heartbeat, Abdominal Circumference (AC), Head Circumference (HC), and Amniotic fluid index (AFI). Then 4D scans record spontaneous baseline movements without any stimuli presentation for approximately five minutes. Next, fetal movements are recorded during the presentation of an auditory habituation paradigm that includes social sound stimuli (‘MA’ and ‘MO’) based on the design by Reissland et al. [13], and pink noise (PN) as a non-social sound stimulus. For each sound stimulus, 1-minute blocks of duplicate presentations by a female voice are played, including identical presentations of the stimuli for 0.4 seconds (+0.8 second silence), with a 5 second silent interval between the blocks. The order of the sound stimuli is counterbalanced and presented in one of the following sequences: ‘MA, PN, MO, PN, MA, MO’, or ‘MO, PN, MA, PN, MO, MA’. The sound stimuli are delivered through speakers placed approximately 60 cm from the mother’s abdomen. The auditory stimuli, containing frequencies between 0.6 Hz and 3.6 kHz, are presented at average decibel levels ranging from 47.6 dB to 54.07 dB, audible to fetuses from 29 weeks of gestational age [47,48].

**Fig 2:**
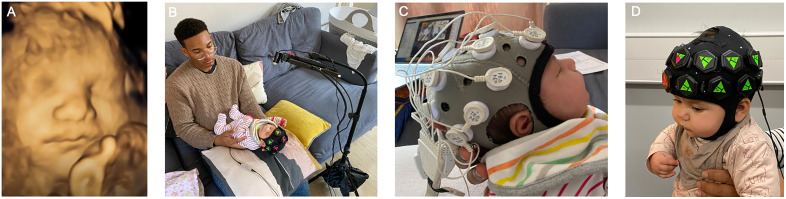
Panel A – 4D ultrasound scan at 34 weeks gestation; Panel B – 1-month-old infant wearing fNIRS headgear, supported by a researcher while sleeping in the home setup; Panel C – 1-month-old infant wearing EEG headgear supported by a researcher while sleeping in the home setup; and Panel D – and 5-month-old infant wearing fNIRS headgear at the research lab visit.

During the 4D ultrasound scanning (Fig 2), we record the fetal face and upper torso (at 24 frames per second), which is later coded offline for fetal movements using an established and reliable coding system for fetal movements [49]. The entire ultrasound procedure takes approximately 30–40 minutes to complete of which 10–15 minutes are dedicated to 4D ultrasound scanning (baseline + in response to habituation paradigm). Both mother and partner (or additional family member) are present in the ultrasound room and are able to watch the fetus via a monitor. The session is adapted to the mother’s needs to ensure she is comfortable throughout. The individuals pictured in Fig 2 have provided written informed consent (as outlined in PLOS consent form) to publish their image.

### Neuroimaging measures

*Functional Near Infrared Spectroscopy (fNIRS)* uses near-infrared light to monitor changes in concentration and oxygenation of haemoglobin in the brain that occur as a result of changes in neural activity. This imaging technique consists of a soft cap where NIR light sources and detectors are mounted and provides several advantages for its use with infant populations such as being silent, relatively portable, and not requiring rigid head stabilization. This allows for use in home and naturalistic settings, improving infants’ and families’ compliance with the study.

In the PIPKIN study, we use a wearable High-Density Diffuse Optical Tomography (HD-DOT) fNIRS system, which uses dense arrays of sources and detectors at multiple separations and with overlapping measurements, allowing for the reconstruction of three-dimensional images with depth information. The HD-DOT system LUMO (Gowerlabs Ltd, UK) consists of multiple independent tiles, each of which is equipped with 3 dual-wavelength LED sources (at 735, 850 nm) and 4 photodiode detectors [50–52]. These tiles can be flexibly clipped into an infant-friendly soft cap to acquire measurements of different brain regions. Our cap for newborn and 1-month-old infants is made from flexible elastane material and features a Velcro chin strap (Easycap GmbH, Germany). It includes 12 tiles positioned over the frontal and temporal regions, providing 36 source locations, 48 detector locations, and a total of 1728 channels per wavelength (Fig 2). For 5-month-old infants, the cap is made of neoprene and includes 15 tiles, offering 45 source locations, 60 detector locations, and a total of 2700 channels per wavelength, covering analogous regions (Fig 2). The sampling frequency of the device is 12.5 Hz.

fNIRS tasks are completed in the home around the first and second week of life and at one month, and then repeated at five months when families are invited to the Cambridge Babylab. The fNIRS paradigms assess a range of cognitive functions and domains, namely social cognition [53], habituation and novelty detection [54] and functional connectivity.

*fNIRS Social/Non-social:* This task consists of dynamic social and non-social videos that are presented consecutively, but not synchronized with auditory social and non-social stimuli. Social videos show a life-size female face moving her eyes and playing games such as peek-a-boo, while non-social videos show a moving toy. Between each condition, as baseline, infants are presented with non-social static images such as cars and trucks. To accommodate infants’ varying awareness states, three variations of this task have been implemented: i) For newborn and 1-month-old infants in awake state, dynamic social videos and dynamic non-social videos are presented with no auditory stimuli [4]. ii) For newborn and 1-month-old infants during sleep, only social and non-social auditory stimuli are presented. Social auditory stimuli include sounds of laughing and crying, while non-social auditory stimuli include sounds of running water, bells or squeaky toys. iii) infants at 5 months perform the full task awake where visual social stimuli are presented alongside auditory stimuli so that a third of the trials contain social auditory stimuli, a third non-social auditory stimuli, and the remaining third are silent ([53], for details of the full task see [55]). Task version i) was discontinued after 97 testing sessions (0–7 day sessions = 28, 7–14 day sessions = 34, 1-month sessions = 35) due to difficulties in acquiring sufficient data from participants. A significant proportion of infants failed to remain awake for the duration of the task, and among those who did, the proportion of infants providing good-quality data was low. The primary challenges included infants becoming fussy and the presence of substantial motion artifacts in the recorded signals, both of which impeded the collection of reliable data. For the remaining testing sessions newborn and 1-month-old infants were tested while asleep according to version ii) above.

*fNIRS Habituation and Novelty Detection* [54]: In this task, infants are presented with the same sentence of around 8 seconds produced by a female voice, repeated fifteen times (i.e., habituation). The next five trials consist of the same sentence, this time produced by a male voice (i.e., novelty). In the last five trials the initial condition (i.e., female voice) is repeated again five times. At newborn visits (including 1-month visit) the infants are asleep, at the 5-month visit an experimenter quietly manipulates toys to keep the infant entertained and in a calm, alert state.

*fNIRS Functional connectivity:* Two tasks are conducted to assess functional connectivity. In newborns and 1-month-old infants, spontaneous task-free hemodynamic activity is recorded during natural sleep for around 20 minutes (to obtain a total of around 10 minutes of motion-free data by combining clean segments, each lasting at least 100 seconds). To improve compliance with the task, infants at 5 months are presented with alternating social (i.e., nursery rhymes) and non-social (i.e., dynamic toys) videos with auditory content of two minutes duration each. Each of these blocks is presented between two and three times.

Where newborn or 1-month-old infants are viewing videos or completing tasks while sleeping in the home, the infant is held in a comfortable position by the experimenter, and the 13-inch screen is placed approximately 30 cm from their face, either attached to a stand, or held in position by a second experimenter (visual angle = 57 degrees). At 5 months, while at the Babylab, all infants are awake for all tasks and for visual tasks are sat on a parent’s lap, 60 cm from a 24-inch presentation screen (visual angle = 54 degrees). Infant behaviour at both sessions is recorded using a webcam attached to the stimuli presentation screen.

The full fNIRS battery, while infants sleep, lasts around 40 minutes for recordings in the home and while awake at 5 months, around 30 minutes at lab visits.

*Electroencephalography (EEG)* is a non-invasive neuroimaging technique that records the electrical activity of the brain and offers high temporal resolution so that rapid electrophysiological changes occurring within a millisecond time-frame can be captured. In the PIPKIN study, EEG is recorded using the 20 channel Neurolectrics Enobio gel-based portable EEG system with passive electrodes with a sampling rate of 500 Hz (Fig 2). The reference and ground electrodes are placed unilaterally on infants’ left mastoid.

*EEG Auditory Oddball:* This task assesses habituation and novelty detection at 1 and 5 months, using the protocol described in Katus et al, [56]. Infants are presented with 15 minutes of auditory stimuli consisting of repeated sounds in randomised order: 80% are frequent sounds (500 Hz pure tones), 10% infrequent sounds (white noise) and 10% trial unique sounds (range of sounds). At all timepoints this task is done while the infant is sleeping. The Auditory Oddball task allows us to examine event-related neural responses to repeated and novel sounds, and to build Bayesian models of the neural computations underlying novelty responses.

*EEG Resting State*: Spontaneous task-free EEG is recorded for around 10 minutes during natural sleep.

*EEG Gaze ERP task:* This visual task completed at 5 months assesses neural sensitivity to eye gaze. For each trial the infant sees a colour image of a female face against a white background on the screen, with the female’s gaze directed either towards or away from the infant. The face then displays gaze shifts, alternating between looking towards and away from the infant. In one third of trials, infants see ‘visual noise’ stimuli which are constructed from the same faces presented in gaze trials, but with phase spectra randomised. For more details of the protocol see Elsabbagh et al [57].

The full EEG battery lasts around 25 minutes for recordings in the home (Fig 2) and about 40 minutes at lab visits.

### Behavioural/Neurocognitive measures

*The Neonatal Behavioural Assessment Scale (NBAS)* is a structured clinical assessment of infant neurology and behaviour, which can be performed from the first few days of life and involves observation of: the infant in both asleep and awake states, responses to stimuli (while asleep), and behaviours relating to social interaction (while awake) such as tracking a moving face, and motor skills and reflexes. It is regarded as the most comprehensive examination of newborn behaviour and requires an initial training period culminating in assessment and certification as described in *The Neonatal Behavioural Assessment Scale* Manual [58]. In PIPKIN, the NBAS is performed during the first two weeks of life, and again at 1 month. The assessment usually takes around 20 minutes, depending on how the infant responds to the assessment and any time needed to calm the infant down.

During the PIPKIN study we amended the NBAS to suit the wider research paradigms being carried out, in order to ensure research sessions were not too taxing for younger infants. In particular, some of the stimuli often used to elicit responses and help babies transition from sleep to awake states, such as reflex items, were removed given infants underwent neuroimaging and other experiences that could elicit such responses. Moreover, certain items were measured and recorded across the whole research session, such as state changes and regulation, with the length of the session documented alongside this so that the number of state changes per 30 minutes (the length of a typical NBAS) could be calculated after the session.

*The Mullen Scales of Early Learning (MSEL)* is a standardised assessment battery that measures cognitive ability and motor development across five domains: gross motor, fine motor, visual reception, expressive language, and receptive language. The MSEL assessment is carried out at the 5-month lab visit, using the standardized protocol appropriate for the age of the infant, as detailed in the Mullen Scales of Early Learning Manual and the Item Administration Book [59].

*Parent-child interaction videos (PCI)* are recorded at 1 and 5 months. The parent is asked to engage in natural play with their child while three video cameras record the interaction; one trained primarily on the infant, one on the parent, and a side view that captures both. At 1 month, a 5-minute play session without toys is recorded in the home with the parent sitting sideways on a sofa (leaning against the arm, their feet up on the seat), with the infant propped up on their knees so they are face-to-face. This position was chosen as a sofa is a constant across varying home environments. At 5 months, a 10-minute recording is made during the lab visit; 5 minutes with no toys, and 5 minutes playing with a standardised set of toys. The parent is seated or kneeling on the floor opposite the infant lying on an angled mat so that they are face-to-face. Where fathers/partners are available during the visit, PCI recordings are made with both parents. Offline PCI videos are later coded to assess multiple aspects of parent and child behaviour, and engagement between the two. Parental interaction style has been shown to associate with child neural and cognitive development [32,60,61].

*Eye-tracking tasks* are carried out at the 5-month lab visit, using the Tobii Pro TX300 remote eye-tracker. The infant sits on the parent’s lap, approximately 40 cm from the 24-inch screen. Following a short calibration sequence, the infant completes four tasks which are interleaved into a single battery which takes around 15 minutes. As some tasks are gaze contingent, the duration of the battery depends somewhat on the infant’s performance.

The eye-tracking battery includes: the *Gap/Overlap task* [62,63] which measures the infant’s ability to disengage from a fixated stimulus, with maturation of the visual system leading to shorter disengagement latencies [64,65]; the *Non-social Contingency task* which examines infant’s sensitivity to contingency and predictability; the *Fifty faces task* (a 40 second long naturalistic video of people going about everyday life, and looking at the camera) and the *Dancing Ladies task* [66] where infants view faces embedded in naturalistic and semi-naturalistic scenes, allowing investigation of visual exploration and face preference as well as standard measures of individual differences such as fixation durations and saccadic reaction time measurements.

### Home environment measures

As well as the experimental data collected at the home and lab visits, we also collect naturalistic measures of the home environment that each infant experiences in a typical day. When the infant is around 3 months old we make a day-long naturalistic recording in the home to capture the infant’s social environment and physiological responses. A researcher visits the participants’ home in the morning to fit the equipment and explain its use. The equipment records throughout the day and the parents remove it at bed/bath time for collection by the researcher the next day. Recording equipment includes: an electrocardiogram (ECG) recording device with built in triaxial accelerometer, a small head-mounted camera to capture the infant’s visual field, a room camera to capture the general space the infant is in, and a LENA (Language Environment Analysis) audio recorder (www.lena.org). During the day, the parent completes a diary recording activities and routines such as feeding and sleeping.

*The ECG and accelerometery* device is purpose-made and records for over 10 hours. It is held in a specially designed cotton baby vest which is worn next to the skin, with the ECG attached using three standard Ag-Cl electrodes in a modified lead II position. To minimise movement of the device and wires, an elasticated cotton wrap is placed over the vest to hold the equipment snugly in place.

*The baby head-camera* made by Babeyes (https://www.babeyes.com) is a small HD smart camera which is clipped to a purpose-adapted cotton hat. The camera measures 5 cm across, weighs 32g and records for 2 hours. Because of the need to minimise the size of the camera, limitations on battery life and storage space mean that it is not possible to use a camera with full-day recording capacity. Parents are provided with a total of four cameras to use throughout the day during periods when the infant is awake. Footage from the camera allows us to identify periods of face-to-face communication and so to calculate a ‘face-time index’ for each infant, giving a measure of the amount of social interaction the infant experiences during a typical day. To compliment this recording and allow us to better understand who is interacting with the infant at times when the infant camera is pointed in a different direction, a room camera has also been introduced into the protocol in the second half of data collection.

*The LENA audio recorder* is held in a purpose-made tabard worn on top of usual clothing, and records the audio environment within 1–3 metres for up to 16 hours. LENA software automatically extracts: 1) Adult word count: the number of adult words the key child hears (whether this speech is directed at the child or not); 2) Key child vocalization count: words or prelinguistic babbling sounds produced by the key child (laughing and crying sounds are not included in this category); 3) Conversational turn count: instances when the key child and an adult speak one at a time in alternating turns. The software can differentiate between live voices and electronic media such as TV or radio. These measures have been shown to be reliable in English [67] as well as in other languages such as Mandinka [68][for a brief review see 69]. In total, we aim to record 7–8 hours of an infant’s home life on a typical day.

**Fig 3:**
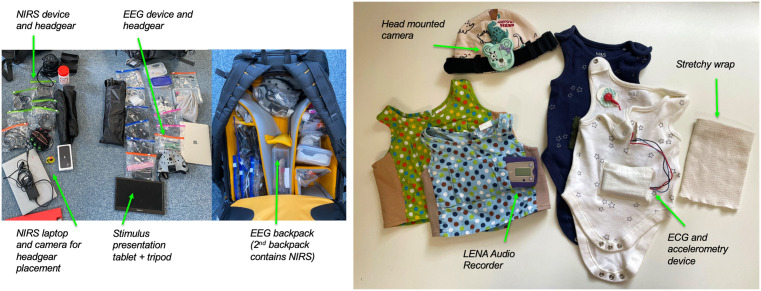
Clothing and equipment used for home recording visits.

The Home Recording Visit clothes are comfortable for the infants and allow participants to behave exactly as they would on a normal day. As the ECG vest is worn next to the skin, and the LENA tabard must be the top layer, infants can be dressed in normal clothing in between. Parents are told that the vest and LENA device can be removed from the infant for comfort when necessary (e.g., during breastfeeding, nappy changing, or when the infant is sleeping or in a car seat), but that the device should be kept close to the infant to continue the audio recording and should remain as the outer layer if additional clothing is put on the infant. Clothing and equipment are shown in Fig 3.

### Questionnaires & interviews – family

Throughout the study participating families complete questionnaires which collect information on family background and lifestyle, parental mental health, maternal diet, feelings and attitudes of parent(s) towards pregnancy and parenting, infant development, and habits and behaviours relating to infant care. Most questionnaires are completed via an online link from REDCap (https://www.project-redcap.org/), but some are asked verbally (indicated by asterisk in Table 3) while the dietary recall utilizes the Intake24 database. For the first phase of the PIPKIN study these questionnaires were sent via email as a link to parents for them to complete at home. While the intention of this was to allow parents to access the questionnaires at a time convenient to them and at their own pace to reduce participant burden, the unintentional consequence was that a significant proportion of families were struggling to notice the email and remember to complete the information without several reminders. This seemed counterintuitive to the intention of reducing participant burden as we were required to contact them more often. Therefore, from February 2024 the timing of the questionnaires was reorganised so that we could conduct these during an existing study time point with participants entering data during the visit using a research tablet. To date, this has significantly improved questionnaire completion and constructively addressed participant burden.

Purpose-designed questionnaires collect demographic, family and household information antenatally and again at 5 and 9 months. This includes information such as household make up, ethnicity, educational attainments, financial status, languages spoken in the home and medical or mental health conditions.

The *Parental Mind-Mindedness recorded interview* [70] is carried out face-to-face antenatally (32 weeks) and at the 5-month lab visit. Parents are interviewed separately and are asked to talk for 3–5 minutes about their child and their (future) relationship with him/her. Recorded responses are later coded to assess the extent to which the parent refers to their child as an individual with their own mind.

The *Expectations About Your Child questionnaire* [71] concerns parental expectations of their child’s character. Parents are asked to imagine the situations described and report in what way they think their child will react in that situation. This questionnaire is administered to both parents antenatally.

The *MacArthur Subjective Social Status Scale* [72] asks participants where they feel their family ranks within society.

*Bringing order out of chaos* [73] measures environmental confusion within the home at antenatal, 1-, 5- and 9-month time points.

The *Adverse Childhood Experience (ACE) Questionnaire* [adapted from 74] asks about possible negative experiences during the mother’s childhood.

The *Brief form of the perceived social support questionnaire* [75] and *Social Exposure* questions probe the family’s wider support network and contact with others both antenatally and across the postnatal period. The *Social Exposure Questionnaire* was devised in-house and assesses social interactions and physical contact experienced by parents over the past month.

Questionnaires probing mental health are sent to both parents and include: The *Positive and Negative Affect Schedule (PANAS)* [76] which asks participants to rate the extent to which they have felt certain positive and negative emotions over the last week; The *Perceived Stress Scale (PSS)* [77] which asks participants to rate how often they have experienced particular stress-related feelings over the last month; the *Hospital Anxiety and Depression Scale (HADS)* [78] and The *Pregnancy Related Anxiety Scale (PRAS)* [79].

At the 5- and 9-month time points both parents complete questionnaires about various aspects of parenting. The *Parental Stress Scale* [80] assesses parents’ feelings about their parenting role, while the *Comprehensive Parenting Behaviour Questionnaire* (CPBQ-0, for infants 3–11 months) [81] contains items of several existing questionnaires assessing parenting behaviour, complemented with newly developed items specifically for parents of young children. The *Maternal Confidence Questionnaire* [82] (referred to as Parental Confidence Questionnaire when applicable to fathers/partners) measures confidence in parenting skills and the parent’s assessment of their ability to recognize the infant’s needs.

The *Adult Temperament Questionnaire* (ATQ) short form is a 77-item self-report questionnaire designed to assess four constructs of temperament: extraversion/surgency, negative affect, effortful control, and orienting sensitivity [83]. ATQ is completed by both parents antenatally, and at 9 months.

The *Assessment of the Day’s Ordinariness* is asked verbally at each testing visit (2 x newborn visits, 1 month and 5 months). This questionnaire asks whether today is usual in terms of the infant’s health, mood and sleep in order to identify effects that may arise as a result of non-experimental factors.

### Questionnaires & interviews – infant

At 1, 2, 3, 4 and 9 months, parents complete questionnaires about their infant’s feeding and sleeping routines. The *Feeding Questionnaire* asks about feeding method (breast/bottle) and feeding routines. The *Sleep questionnaire* combines the *Brief Infant Sleep Questionnaire* [84], the *Sleep and Settle Questionnaire* [85], and additional questions about noise levels in the home environment. The partner version includes a subset of items from the Sleep and Settle questionnaire asking how much the parent is bothered by the infant’s sleep routines, and reasons the infant is unsettled.

The *Infant’s life and routines questions* ask about who primarily cares for the infant across the week, as well as screen time and language exposure.

*The Infant Behavioural Questionnaire (very short form)* (IBQ-R VSF) [86] measures infant temperament across three scales: positive affectivity/surgency, negative emotionality, and effortful control.

The *Infant/Toddler Sensory Profile* [87] measures an infant’s sensory processing abilities and the effect of sensory processing on the infant’s functional performance in daily life.

The *UK Communicative Development Inventory* [88] is used to assess language development at 9 months. This consists of a vocabulary checklist where parents indicate which words their child can understand, and which they can both understand and say.

The *Vineland Adaptive Behavior Scales* [Vineland-II; 89], provides a standardised measure of adaptive behaviour. Scores from the four domains (Communication, Daily Living, Socialization, and Motor Skills) can be combined to create an Adaptive Behavior Composite (ABC) score. The adaptive nature of the questionnaire is built into the logic of the online questionnaire platform so that the next item is determined by the responses to previous items.

### Medical and health details/sample collection

The *Healthy Pregnancy Questionnaire* is asked verbally at the first postnatal home visit, and covers details of the pregnancy and birth, current health status of mother and baby, and lifestyle questions such as smoking, alcohol consumption, and weight gain during pregnancy.

For the *Maternal Diet Diary* mothers are asked to record their diet for a 24-hour period using the Intake 24 app during the third trimester of pregnancy, and again when their infant is 1 and 9 months old. This is a free, open-source dietary assessment research tool, provided, maintained and developed by the Cambridge NIHR Cambridge BRC Measurement Platform at the MRC Epidemiology Unit, University of Cambridge.

*Hair samples for cortisol analysis* are collected at three time points for mothers (antenatally at around 34 weeks and at the 3-month and 5-month timepoints) and are taken by the experimenter. The hair is taken from the posterior vertex from the scalp and is cut as close to the scalp as possible. The sample is approximately the thickness of a penny at the base (~10 mg). The hair is then tied at the scalp end (in order to delineate which end is which) and stored.

*Nail samples from the infant* are collected by parents via instructions provided by the experimenter at around 3 and 5 months. Samples are placed in a collection box which is collected by researchers at the 3-month and 5-month timepoints. These collection boxes are labelled to detail the exact date on which the nail clippings were obtained.

For analysis, hair samples (∼10 mg) are placed in borosilicate tubes, washed twice in isopropanol and dried for one day. Samples are cut inside the borosilicate tubes into 1–3 mm pieces using surgical scissors, and then mixed with methanol and incubated in a sonic bath for 4 hours before being removed and placed on a shaker for one day. Samples are then centrifuged at 4000 rpm at 4 °C for 10 min. The dry extract is reconstituted in a standard assay dilution buffer and frozen.

Finally, cortisol assay HCC (hair cumulative cortisol) or NCC (nail cumulative cortisol) is quantified using previously validated and commercially produced ELISA kits designed to measure free cortisol in human saliva (Salimetrics, Inc., State College, PA). Twenty-five microliters of reconstituted sample are then run in duplicate as directed by the assay protocol. Assay output in ug/dL is converted to pg/mg.

During feasibility work we first piloted the collection of samples for cortisol analyses across a wider range of parameters, including more regular data collection points from birth to five months of age and hair sample collection for partner and infant in addition to the mother. Furthermore, we ran feasibility analyses to calculate the sample weight required for the fingernail samples from the infant. This pilot work revealed several challenges which led to an adaptation of the protocol in its current version. The hair sampling from infants and partner proved difficult due to the often lower availability of hair in young infants of this age, the lower participation rate of partners (either due to low participant engagement or low volume of hair preventing sampling) and the low number of fingernail samples available for infants (either because fingernails broke off and were lost, because parents found it challenging to collect samples or because they often forgot to carry this out at the frequent number of timepoints initially trialled). While we continue to collect fingernail samples from the infants to test the feasibility of extracting cortisol from infants, we have reduced the number of timepoints to two and furthermore expect the sample size of this part of the project to be lower and used for feasibility analyses only.

### Data analysis

Due to the large amount of data generated by the PIPKIN study over a number of timepoints and using a variety of methods, it will take some time process the data and publish the final results. Data collection will conclude by 1^st^ March 2026, and it is expected that results will be published from later that year.

### Ultrasound measures

Ultrasound data is coded frame-by-frame using offline video recordings in the Observer 15XT software programme (Noldus, the Netherlands) employing Fetal Observable System Coding Scheme (FOMS) [13] to analyse fetal behaviours, such as orofacial movements, touch movements, head movements, and observable eyeblinks. The coding process includes: i) observing each scan at full speed to become familiar with the neutral face, determine the fetal state [active/quiet, sleep/awake;, [90], and calculate the codable length of the area of interest; ii) coding the total codable footage of the scan frame-by-frame from the upper torso to the lower face and upper face respectively; iii) classifying facial gestalts based on the type (e.g., cry-face, laughter-face) and complexity (based on the number of movements that occur simultaneously); iv) calculating the relative frequency of each distinct movement and gestalt per minute to account for variations in scan length; v) measuring the duration of each movement and gestalt. All scans are coded by expert coders trained in coding 4D scans and facial expressions. To ensure consistency of codes across time and among different coders, inter- and intra-reliability checks will be applied to 10% of the ultrasound data.

### Neuroimaging measures

The fNIRS and EEG data will be pre-processed and analysed to identify statistically significant regions of brain activation, from which markers of brain function in relation to specific tasks will be extracted. The resulting data will be used to generate measures of brain function for age. Multivariate data analyses will enable the exploitation of longitudinal and cross-sectional variation in testing-age to fit growth curve models. These are valuable for providing a framework for efficient age-adjusted comparisons of differences in average trajectories according to socio-economic status, groupwise differences in variability, and also for characterizing the range of individual developmental trajectories.

*fNIRS functional connectivity:* The analysis of fNIRS functional connectivity data will involve different methods to describe functional brain networks and their developmental trajectory. One such method is the seed-based correlation approach (e.g., [91]), which involves selecting a region of interest as a seed and computing the correlation coefficient between the seed and all other brain regions. Another approach will use group-level independent component analysis with dual regression to compute spatially independent patterns of brain activity (e.g., [51, 92]). This method generates a set of group-level maximally independent spatial maps (i.e., functional networks) that can be regressed out to the subject level using spatiotemporal or dual regression to obtain subject-specific spatial maps [93]. These individual-level maps can be used to assess individual trajectories of functional connectivity development.

The primary goal of these methods would be to describe functional brain network development during the first weeks of life and study interactions between pre- and postnatal risk factors, emerging social and cognitive skills, and atypical developmental trajectories in functional connectivity. We aim to describe functional connectivity at different time points and use connectivity metrics to understand whether these could be predictive of individual cognitive/behavioural performance and variability. We also plan to investigate common organizational principles and stable individual features of functional networks across task-free and task-based paradigms, assessing variations due to task state. This approach will allow us to establish individual-specific connectivity profiles that can potentially be used as predictors of individual differences in cognition and behaviour.

*ERP analyses:* This study employs EEG event-related potential (ERP) tasks at 1 and 5 months to examine early neurodevelopmental trajectories using auditory and gaze-based paradigms, drawing on approaches from Katus et al. [56] and Elsabbagh et al. [62]. The auditory ERP analysis aims to examine habituation and novelty detection, assessing developmental shifts from intensity-driven to novelty-driven responses by measuring P3, Nc, and early components (N1, P1, N2). Based on evidence from Katus et al. [56], intensity-based P3 responses at 1 month are expected to transition to novelty-driven responses at 5 months, with stronger habituation correlating with better cognitive outcomes. The gaze ERP task assesses face orienting and processing via first-look preferences, total looking time, and ERP components (P1, N170/N290, P3, Nc), with predictions that early face biases and eye gaze processing at 1 month become more specialised at 5 months, supporting emerging social brain expertise [62,94].

*EEG resting state:* For the resting-state EEG analysis, data collected at 1 and 5 months will be used to examine functional connectivity and frontal alpha asymmetry (FAA). Functional connectivity will be measured using common methods, such as coherence, phase-locking value (PLV), or weighted phase lag index (wPLI). The maturation of connectivity patterns will be assessed between 1 and 5 months. Frontal alpha asymmetry will be analysed by comparing log transformed power in the left and right frontal areas, which may indicate early tendencies toward approach or avoidance behaviours. Stability of membership in the relative left or right frontal asymmetry groups will be assessed between 1 and 5 months.

In summary, neuroimaging data will be analysed via multiple approaches to investigate the development and variability of ERP responses and functional brain networks across the early stages of life, with the goal of identifying associations with specific and general environment factors.

### Behavioural/Neurocognitive measures

Each of the behavioural measures will be analysed according to predefined scoring systems, returning a normalized quantitative score for each infant (and where applicable, each parent, i.e., maternal interactions during PCI identified through video coding) at each age point. All questionnaire data will be analysed using standard methods according to the scoring system of each individual questionnaire.

### Home environment measures

LENA recording data will be analysed to give: Adult word count, infant vocalisation count, and number of adult-infant conversational turns. The face-time index will be calculated using the head camera to determine the proportion of recording time that the infant experiences face-to-face communication during a typical day. Infant heart rate and activity will be linked to the face-time index to examine physiological responses to social communication.

### Cortisol

Cortisol levels from hair and fingernail samples will be analysed to determine 1) whether maternal hair cortisol is significantly associated with infant neurocognitive outcomes (i.e., performance in task based fNIRS and EEG), 2) whether infant nail cortisol is a viable method of measuring infant cortisol soon after birth and 3) the stability of maternal hair cortisol levels pre- and post-birth. Furthermore, analyses will explore whether associations found for [1] are mediated by maternal mental state, pregnancy-related anxiety and birth-specific factors (delivery type, complications), and how maternal psychological stress accounts for variability in fetal behaviour and in offspring cortisol levels immediately following birth and the trajectory of cortisol in the subsequent months.

### Analyses and data availability

To study inter and intra-individual differences, analyses will involve approaches that allow assessment of the full cohort, as well as statistical models accounting for typical variation in available data to assess the contribution of early adversity/contextual risk factors to individual infant development. To maximise power, longitudinal multivariate analyses will make use of full information maximum likelihood setups that use both complete and partially complete data records, and where appropriate the multiple assessments of the same construct. Typical MANCOVA style testing will be undertaken in a generalised structural equation modelling framework (SEM/mixed models) suitable for (i) analysing both normally and non-normally distributed infant measures, (ii) the study of longitudinal and cross-sectional variation in testing age to fit growth curve models and (iii) comparison across different measures. Whenever possible, comparison data from previous cohorts will be included for combined or replication analyses.

The four study hypotheses will be addressed as follows:

*Hypothesis 1*: Inter-individual differences in basic sensory and motor processing are consistent between late prenatal and early postnatal periods.

This hypothesis will be tested by first assessing fetal movement in response to social and non-social habituation paradigms at 32 and 34 weeks gestation. We expect that at each gestational week, fetuses will react differently to social and non-social auditory stimuli (e.g., greater velocity of movement; displaying mimicry orofacial reactions to social auditory stimuli), with a decrease in arousal, movement velocity and an increase in habituation upon repeated exposure. Also, we expect to observe developmental changes in fetal auditory habituation rates between 32- and 34-weeks’ gestation due to maturation.

Second, we will investigate the relationship between these fetal habituation rates and postnatal habituation measures using data from the Neonatal Behavioural Assessment Scale (NBAS) and the fNIRS Habituation and Novelty Detection (HAND) oddball task which are carried out during the newborn period. Lastly, we will assess spontaneous rhythmical movements such as hand-to-mouth and arm movements, comparing baseline spontaneous movements pre-birth with post-birth measures recorded during NBAS and PCI.

Exploratory analysis of later postnatal outcome measures will also be conducted, to assess whether inter individual differences in fetal auditory habituation rates are related to observable differences in postnatal assessments at 3, 5 and 9 months.

*Hypothesis 2:* There is rapid change in cortical functions over the first postnatal days, particularly for brain networks that support social behaviour.

Hypothesis 2 will be assessed by determining change between brain function (NIRS) measurements during the first and second weeks, and at one month. Specifically, in accordance with the Interactive Specialisation model of emerging cortical functions [95] we will assess developmental trajectories of both the spatial specificity and magnitude of the observed cortical responses. For the *fNIRS Social/Non-social* task version (i) (visual only) we predict stronger haemodynamic responses to social dynamic stimuli vs non-social dynamic stimuli over posterior superior temporal sulcus and temporoparietal junction channels. For version (ii) (auditory only) we predict a more spatially restricted vocally selective (vocal > non-vocal) response over the anterior superior temporal gyrus and middle temporal gyrus channels during the neonatal period, which will become stronger with age. Furthermore, we predict more widespread spatially dispersed non-vocally selective response at the youngest ages which will specialise towards vocal selectivity across the first weeks and months of postnatal life [95]. For version (iii) (both visual and auditory) we expect both these effects.

For the fNIRS *Functional connectivity task* we expect intra hemispheric connectivity to be stronger than inter hemispheric and anterior-posterior connectivity during the first week(s) of life with a shift to more long-range connectivity as the infant becomes older. We will explore whether those infants with more rapid developmental trajectories of connectivity patterns associate with those showing more rapid social selectivity during the *fNIRS Social/Non-social* task.

We will also assess continuity and change in the social interaction and visual orienting measures from the Neonatal Behavioural Assessment Scale (NBAS) between newborn and 1-month timepoints, and assess how these behavioural responses correlate with the fNIRS measured brain responses.

Behavioural measures taken at later study timepoints such as eye tracking and Mullen (5 months), and Communication Development Inventory (CDI) and Vineland (9 months) will be used to assess whether differences in early brain development relate to observable differences in behaviour in subsequent months.

*Hypothesis 3:* Subsequent development in brain function is associated with experience of social interaction with other humans.

Hypothesis 3 will be assessed by initially determining changes in brain function (NIRS) measurements between one month and at five months, again assessing developmental trajectories of both the spatial specificity and magnitude of the observed cortical responses [95].

Similarly, we will use EEG data collected at 1 and 5 months to examine developmental changes in emerging spatial and temporal specificity of activation in response to auditory stimuli, and will also assess how this relates to individual differences in sensory processing.

Furthermore, these measures of brain function will also be related to measures of social experience to assess how experience of social interactions influences the emergence of frontal and temporal cortical social brain function [94]. Measures of social experience include the ‘face-time index’ calculated from the baby head-camera footage during the home recording visit at 3 months, the amount of language the infant is exposed to in a typical day (as recorded by the LENA recorder during the home recording visit), caregiving practices (recorded via questionnaires), and measures of parent sensitivity and responsivity coded from the Parent-Child Interaction videos at 1 and 5 months.

*Hypothesis 4:* Individual differences in developmental trajectories associate with low resource (poverty) associated risk factors.

We aimed to recruit participants from diverse backgrounds, particularly from less affluent areas and rural settings (i.e., those less likely to participate in research). The questionnaires that parents complete throughout the study will give us a rich understanding of each child’s home environment, including factors associated with low-resource settings such as parental education levels, family income, multiple deprivation index of home postcode, organisation of home life, social and family support, and mental health and wellbeing. These measures will allow us to investigate differences in the measures and analyses described above that relate to early exposure to poverty.

Furthermore, we will assess whether physiological factors relating to stress, such as higher mean heart rate, lower heart rate variability and higher levels of cortisol vary between infants from high- and low-resource families.

Upon completion of data curation, details of data availability will be published on our Open Science Framework page.

## Conclusions

The PIPKIN study is a comprehensive and multi-method study of development during pregnancy and the first 9 months of postnatal life in the UK, with a particular focus on the perinatal transition from pre- to post-natal life. The combination of neuroimaging, behavioural, parent-report measures, and biological samples provides a unique opportunity to study a variety of context-associated moderators which may or may not be related to poverty, the social environment and family context, as well as the mechanistic processes underlying associations between markers and outcomes. On one hand, we hope that our work will be an asset to the study of neurocognitive development in early infancy, particularly the use of still emergent neuroimaging tools. On the other hand, this work has broader value for developmental research in general. Due to the logistical and financial constraints of longitudinal research, our project is among the few to assess early development across a large number of study visits starting during pregnancy, and to incorporate such a variety of methods. Thus, the generated results will enable us to identify critical windows for developmental vulnerability and act as rationale to guide future interventions which aim to protect and enrich the developing brain within contexts associated with risk. We propose that our project provides a roadmap for other researchers interested in conducting studies of neurocognitive development in more naturalistic home environments with similar contextual factors.
